# Strategic Self-Talk Assists Basketball Free Throw Performance Under Conditions of Physical Exertion

**DOI:** 10.3389/fspor.2022.892046

**Published:** 2022-06-17

**Authors:** Evangelos Galanis, Antonis Hatzigeorgiadis, Fedra Charachousi, Alexander T. Latinjak, Nikos Comoutos, Yannis Theodorakis

**Affiliations:** ^1^Department of Physical Education and Sport Science, University of Thessaly, Trikala, Greece; ^2^School of Social Sciences and Humanities, University of Suffolk, Ipswich, United Kingdom

**Keywords:** shuttle run, exhaustion, self-talk mechanisms, attention, applied sport psychology

## Abstract

The purpose of the present study was to examine the effects of a strategic self-talk intervention on basketball free throw performance under conditions of physical exertion. Forty-one male basketball players (Mage = 23.19 years) participated in the study. Following a baseline assessment, a 3-week intervention was implemented. During this period participants of the two groups practiced the same number of free throws in their training sessions; participants of the experimental group practiced using self-talk and developed personal free throw self-talk plans. In the final assessment, the participants repeated the free throw test following a typical shuttle run task causing increased physical exertion. The results showed that in the final assessment the self-talk group performed significantly better than the control group. Overall, the findings indicate that self-talk can be an effective strategy for basketball players when performing free throw under conditions of physical exertion, which is a typical situation in basketball games.

## Introduction

Applied sport psychology practice posits a central position in the knowledge map of sport psychology (Latinjak and Hatzigeorgiadis, 2021), as mental strategies for the development of mental skills and performance enhancement are the active ingredients in the provision of sport psychology services. Within such strategies, self-talk interventions have been receiving increasing research attention in recent years. Strategic self-talk involves the development and use of predetermined self-talk cues or plans that can serve self-regulation purposes and enhance performance (Latinjak et al., 2019).

The vast majority of self-talk research has focused on the effectiveness of strategic self-talk in enhancing performance in sport tasks. Such studies have been implemented in different settings, laboratory and field, involving a diverse array of sport tasks, such as fundamental motor skills (e.g., vertical jump, Edwards et al., 2008), features of sport performance (e.g., tennis stroke, Cutton and Landin, 2007), and endurance tasks (e.g., swimming endurance performance, de Matos et al., 2021). Overall, the significant impact of strategic self-talk interventions in sport has been evidenced through a systematic review supporting the performance facilitating effects of self-talk (Tod et al., 2011), and a meta-analysis providing evidence for a medium effect size, and identifying moderators that can explain differences in the effectiveness of self-talk (Hatzigeorgiadis et al., 2011).

In particular with regard to physical exertion, several studies have provided evidence for the effectiveness of strategic self-talk. Blanchfield et al. (2014), using a time to exhaustion protocol in a cycling task found that motivational self-talk enhanced time to exhaustion from pre-test to post-test. Similar findings have been reported by Barwood et al. (2015) in a 10 km timed trial. Even though these studies assessed performance in endurance tasks (rather than fine tasks while physically exerted), the effectiveness of self-talk was attributed, based on the psychobiological model of endurance performance (Marcora et al., 2009), to participants' lowered perceived exertion. This evidence suggests that self-talk may have an important self-regulation role under conditions of physical exertion. Further research has explored the value of self-talk for self-regulation purposes, studying the effects of strategic self-talk on anxiety and volitional skills (Walter et al., 2019), self-efficacy (Hatzigeorgiadis et al., 2007), and attention (Galanis et al., 2021). Such research has supported the self-regulatory function of strategic self-talk and, in addition, has helped understanding the mechanisms through which self-talk facilitates task performance.

A recent line of research has been targeting the effectiveness of self-talk for performance enhancement and self-regulation under adverse conditions. Many times, the athletes have to perform under stressful conditions, which may influence various physiological (e.g., heart rate, oxygen consumption) and psychological (e.g., anxiety, anger) aspects, but also coordination and attention. In sport contexts, adverse conditions may be consisting of extreme environments (e.g., hot or windy weather), distracting situations (e.g., interfering thoughts or crowd movements and noises), and increased loads (e.g., physical and mental fatigue). The ability to overcome such condition, and maintain efficiency and commitment despite the adversities is an important endeavor. Toward this direction, the use of cognitive strategies in general and self-talk in particular may be of particular importance.

With regard to environmental conditions, Hatzigeorgiadis et al. (2018) examined the effects of strategic self-talk on a 30-min cycling task in strenuous conditions of elevated heat. Findings revealed that the self-talk group sustained a steady power output throughout the duration of the task, whereas the power output of the control group decreased for the last 10-min of the task. In a similar study in heat condition, Wallace et al. (2017) supported that following a two-week intervention programme strategic self-talk improve endurance capacity in a cycling task but also cognitive functioning.

Considering the impact of distractions, Galanis et al. (2018) tested the effects of self-talk on task performance under conditions of external distraction, introduced by loud, intermittent noise, in laboratory and field settings. For the laboratory experiment, they reported that the self-talk group performed better than the control group on a computerized test requiring focused attention, whereas for the field experiment involving free throws in basketball they reported that players of the self-talk group performed better than athletes of the control group.

Finally, considering mentally challenging states, Gregersen et al. (2017) tested the effects of self-talk strategies under condition of ego depletion on a selective attention computerized test. They found that self-talk in the form of attention-alerting and attention-directing cues led to faster reaction times and greater percentage of correct responses on the task compared to the control group. This experimental design was subsequently tested a sport task. In particular, Galanis et al. (2022) conducted an experiment testing golf-putting performance under conditions of ego depletion. The results showed that following a self-talk intervention putting performance for the experimental group improved significantly, whereas that of the control group did not change. Collectively the above findings suggest that self-talk can counter the negative consequences that are caused by decreased strength of self-control (Baumeister et al., 2007).

Taking into consideration the evidence regarding the effectiveness of self-talk in general, and under adverse conditions in particular, the present study aimed to further extend this research line. Accordingly, the purpose of the present study was to examine the effects of a strategic self-talk intervention on basketball free throw performance under conditions of elevated physical exertion. We hypothesized that the strategic self-talk intervention would assist free throwing performance in physically fatigued basketball players.

## Methods

### Participants

Power analysis (G^*^Power, Faul et al., 2007) based on the meta-analytic effects of strategic self-talk on sport tasks (*d* = 0.48; Hatzigeorgiadis et al., 2011), showed that to achieve a power of 0.80 at an alpha of 0.05, a total of 37 participants were required. Forty-one male basketball players from four different teams, two from first regional division and the other two from second regional division, participated in the study. The teams from each division were randomly assigned as experimental (*n* = 22 basketball players) and control (*n* = 19 basketball players). The mean age of the players was 23.19 (*SD* = 6.42 years).

### Procedure and Intervention

The institution's ethics committee provided permission for the conduct of the study (ref: 1378). The experiment included a baseline assessment, an intervention period and a final assessment. In a preliminary meeting, all participants received instructions regarding the requirements and the procedures of the experiment. They were also informed that participation was voluntary and that they could withdraw from the study at any time. Subsequently, they signed a consent form.

#### Baseline Assessment

At the start of the session, participants received instructions regarding the baseline assessment. Each player had to shoot eight sets of free throws in pairs (16 free throws in total), as free throws in basketball games are most often performed in pairs. The baseline assessment took place after their warm up. Before the onset of the assessment participants completed a 6-item questionnaire assessing states of physical fatigue, including cardiovascular exertion (I feel tired, I feel exhausted, I feel I run out of energy) and muscular (my legs feel weak, my leg muscles are aching, my legs are tired) fatigue, on a 10-point scale (from 1 to 10; not at all to extremely) based on Hecimovich et al. (2014) scale.

#### Intervention Program

The duration of the experimental intervention was 3 weeks and the frequency of delivery was three times per week. In each practice session participants performed eight sets of free throws pairs after their warm up session. Players of the experimental group were introduced in the use of strategic self-talk through a short presentation. At the beginning of each training session, just before the onset of the scheduled sets, they were receiving instructions about self-talk plans (what to say, when to say, why to say it) (Hatzigeorgiadis et al., 2014). The purpose of the intervention program was (a) to educate players on the use of self-talk, (b) to get them to train using self-talk consistently, and (c) to enable them to develop personal self-talk plans for their free throws. During week 1 players practiced using instructional self-talk cues (e.g., “elbow,” “target,” “knees,” etc.); during week 2 they practiced using motivational self-talk cues (e.g., “I can,” “in,” “ready,” etc.); finally, during week 3 they developed their own free throw self-talk plan for the final assessment. Upon completion of each free throwing session participants were asked to verbally report how frequently they were using the self-talk cues during the execution on a 10-point scale (1 = not at all, 10 = throughout the set).

Players of the control group received a short presentation regarding the importance of free throws in games. For the training, similarly to the experimental groups, they were performing eight sets of free throws following their warm-up.

#### Final Assessment

The final assessment included two phases: the induction of physical fatigue and the free throw shooting. First, the shuttle run task was performed to induce physical fatigue. The task involves continuous running between two lines 20 m apart in time to recorded beeps, with the time between recorded beeps decreasing each minute. The number of runs completed within the set beeps was recorded. Upon completion of the run and just before the start of the final assessment players were asked to orally indicate their perceived exertion on the 6–20 Borg scale (RPE, Borg, 1998) and completed the 6-item questionnaire assessing states of physical fatigue; this procedure lasted between 40 and 60 s.

Immediately after the completion of the shuttle run players were asked to perform eight sets of free throw pairs. Players of the experimental group were instructed to use their personal self-talk plan they developed during the intervention program. After the completion of the final assessment, all participants completed a typical self-talk manipulation check protocol (Hatzigeorgiadis et al., 2009).

## Results

### Control Measures and Baseline Differences

#### Baseline Differences

An independent sample *t*-test showed no significant differences between the two groups in the percentage of successful free throws for the baseline assessment, *t*_(39)_ = 0.10, *p* = 0.92.

#### Physical Fatigue Manipulation

A one-way multivariate analysis of variance was performed to test for differences in completed runs and RPE upon completion of the shuttle run between the two groups. The analysis showed non-significant multivariate, *F*_(3, 37)_ = 0.96, *p* = 0.42, and univariate effects; for completed runs, *F*_(1, 40)_ = 0.15, *p* = 0.71, and RPE, *F*_(1, 40)_ = 2.98, *p* = 0.09.

A mixed model two-way ANOVA with one repeated factor (condition) and one independent factor (group) was performed to test for differences in physical fatigue between baseline and final assessment as a function of group. The analysis showed a significant condition effect, *F*_(1, 39)_ = 93.25, *p* < 0.001, partial η^2^ = 0.71, and a non-significant condition by group interaction, *F*_(1, 39)_ = 0.03, *p* = 0.87, indicating that physical fatigue increased comparably for both groups. Descriptive statistics for control measures and manipulation checks are presented in Table 1.

**Table 1:** Descriptive statistics for control measures and manipulation checks.

	**Control**	**Experimental**
Baseline performance (% of successful free throws)	62.53 ± 17.69	63.05 ± 16.87
Shuttle Run–completed runs	58.89 ± 26.54	61.68 ± 20.33
RPE–post-run (range 6–20)	16.47 ± 1.39	17.27 ± 1.55
Physical fatigue–Baseline (range 1–10)	3.02 ± 1.77	3.28 ± 1.61
Physical fatigue–Final (range 1–10)	6.33 ± 1.84	6.72 ± 1.86

#### Use of Self-Talk in Training and Final Assessment

Players of the experimental group reported consistent use of self-talk during the training sessions across the intervention (*M* = 8.42, *SD* = 0.76); the mean scores for the 3 weeks were respectively: 8.32 ± 1.32; 8.27 ± 1.06; 8.76 ± 0.92. Similarly, for the final assessment they reported consistent use of self-talk (*M* = 8.86, *SD* = 1.17); no player from the control group reported consistent use of strategic self-talk during the experimental testing (based on the recommendations of Gregersen et al., 2017).

### Free Throw Performance

One-way ANCOVA was performed to test for performance differences under conditions of physical exertion, while controlling for performance in the baseline assessment. The analysis showed a significant group effect, *F*_(1, 41)_ = 35.61, *p* < 0.001, partial η^2^ = 0.48, with the estimated means showing that performance of the experimental group (*M* = 69.33, *SE* = 1.98) was better than that of the control group (*M* = 51.98, *SE* = 2.13).

**Figure 1:**
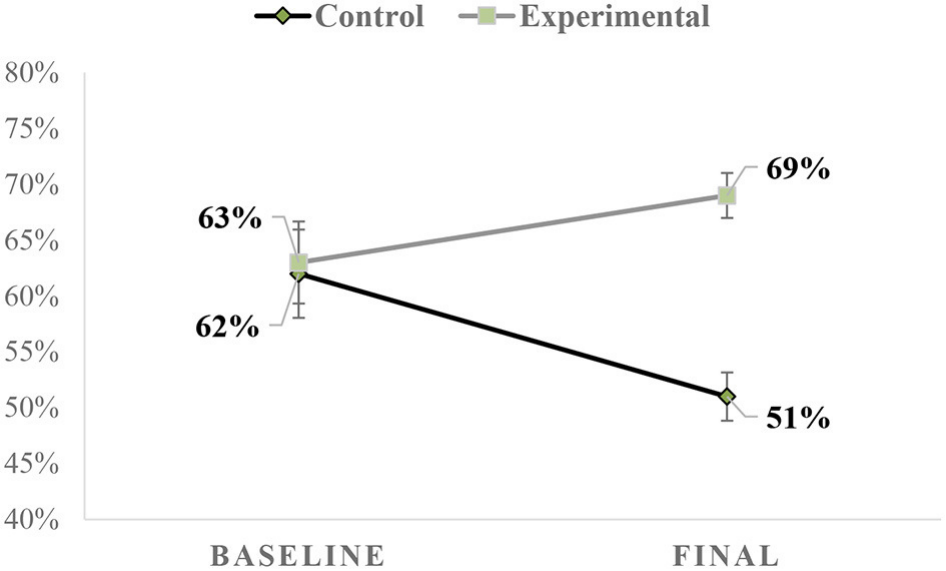
Mean scores for free throw shooting performance (percentage) at baseline and final assessment.

Additionally, a mixed model two-way ANOVA with one dependent factor (condition: baseline, final) and one independent factor (group: control, experimental) was performed to examine for changes in performance as a function of the induced physical exertion. The analysis revealed a significant group by time interaction, *F*_(1, 39)_ = 9.43, *p* = 0.004, partial η^2^ = 0.20. Examination of the pairwise comparisons per time revealed that (a) for the control group percentage of successful free throws decreased significantly from baseline to final assessment (*p* = 0.012) and (b) for the experimental group there were no significant differences between the two measures (*p* = 0.100). The mean scores for the two groups at baseline and final assessments are presented in Figure 1.

## Discussion

The purpose of the present study was to examine the effects of a self-talk intervention on basketball free throw performance, a task requiring composure, under conditions of physical exertion that resemble the field conditions. Findings showed that the use of self-talk by the experimental group led to better performance in free throws, compared to the control group. The findings support that self-talk can be an effective self-regulation, performance enhancing strategy under physically demanding conditions.

In order to ensure the integrity of the experiment, several manipulation checks were performed. First, the effectiveness of the physical fatigue manipulation was evaluated. Physical fatigue increased similarly for both groups from baseline to final assessment. For the final assessment, players reported a considerable level of RPE and, accordingly, cardiovascular and muscle fatigue, while no differences were recorded between the two groups. This confirms that the manipulation was successful in inducing the required conditions and, in combination with the lack of baseline differences on free throw performance, to ensure that the differences identified in the final assessment were not due to individual differences in free throw shooting ability or physical condition. In addition, players of the experimental group reported an adequate use of self-talk throughout the training and the final assessment, that supports that through the self-talk training players managed to maintain consistent use of self-talk. Overall, the findings of the manipulation checks support the integrity of the experimental conditions and thus the internal validity of this field experiment.

The study followed a recent research line exploring the effectiveness of self-talk under adverse conditions. The findings further extend this literature providing evidence for the beneficial effects of self-talk on an attention requiring field task under conditions of physical exertion. Research on the impact of physical exertion on cognitive processes has provided inconsistent findings. Evidence have shown that moderate intensity exercise may have a positive effect on cognitive task performance (e.g., Kumar et al., 2012); however, physical exhaustion has been linked to reduced response accuracies in cognitive tasks (Itagi et al., 2018). Overall, the relationship between exercise and cognitive performance has been characterized as complex (Lambourne and Tomporowski, 2010) and may differ as a function of timing (during or following exercise), exercise modality (e.g., cycling vs. running) and intensity (moderate vs. vigorous). In sport, over the course of training or competition athletes are required to make choices and decisions while physically exerted and mentally fatigue. In a field study with netball players, physical fatigue was positively related with the number of turnovers (Russell et al., 2020).

In the present study, under conditions of physical exertion, performance of the experimental group was superior to that of the control group. Considering the demands of the task, free throw shooting in basketball is characterized by precision and requiring high levels of composure and focused attention. Even though attentional aspects were not assessed in this study, it can be speculated that strategic self-talk assisted performance of the experimental group through maintaining or enhancing attentional functions. Such speculation can be based on previous findings supporting the attentional effects of strategic self-talk (e.g., Galanis et al., 2018, 2021). Thus, the study provides in field indications that the attentional effects of self-talk may be a viable mechanism explaining the facilitating effects of self-talk on performance. Two interrelated but seemingly different interpretations have been suggested to account for such a postulation. The first interpretation is that self-talk can help diminishing the potential impact of exertion. Galanis et al. (2018), in a similar study in the field with basketball players taking a free throw test, reported that self-talk could counter the impact of external distractions and increased percentage of successful free throws. The second interpretation is that self-talk can help enhancing the effectiveness of focused attention required, thus improving performance in fine tasks. Galanis et al. (2016) provide considerable evidence for the facilitating effects of self-talk on the different attention functions, even though these effects were not under physically or mentally demanding conditions. The present study provides evidence regarding the effectiveness but also the potential mechanisms of self-talk in a sport setting and encourage further research toward this direction.

An aspect of the study that requires caution involves the design. To confidently claim the impact of fatigue on free throw performance, a neutral control group (i.e., no strategic self-talk intervention, no physical exertion condition) would be required. Considering that field character of this experimental study and the competitive athlete samples that were employed, which restrict methodological choices that can be made, we opted for a two-group study and decided to have different conditions from baseline to final assessment that would provide at least indications regarding the impact of our physical exertion manipulation. Even though the shuttle run protocol induced the desired fatigue, this does not ensure that performance decreases for the control group were due to this fatigue, as, for example, motivational losses may have occurred in the control group. Although a design with an additional, neutral group would strengthen our confidence in the results, we feel that decrements in the performance of the control group provide reasonable indications for the negative impact of induced fatigue on performance in this basketball task.

Overall, the study provides valuable empirical evidence and holds, we believe, important external validity. Participants were competitive basketball players, assessed on a typical basketball skill, which is an important feature of the basketball game altogether (Kozar et al., 1994). In addition, considering that basketball is quite a physical sport and that in-between intense moments players have to compose themselves and execute successfully free throws, the condition that was induced should be considered similar to the realistic settings, thus further enhancing the ecological validity of the experiment. Accordingly, basketball players and coaches should be strongly encouraged to adopt and train systematically self-talk in plain but also in physically and mentally demanding conditions to enhance players' success in free throws.

## Data Availability Statement

The raw data supporting the conclusions of this article will be made available by the authors, without undue reservation.

## Ethics Statement

The studies involving human participants were reviewed and approved by Bioethics Committee, Department of Physical Education and Sport Science, University of Thessaly. The patients/participants provided their written informed consent to participate in this study.

## Author Contributions

EG participated in the development of the intervention, prepared the manuscript, supervised the data collection, and analyzed the data. AH conceived the study, developed the methods, contributed to the writing of the manuscript, and reviewed it. FC implemented the intervention and provided feedback on the manuscript. AL helped the development of the intervention and reviewed the manuscript. NC contributed to the development of the methods, assisted the data analysis, and reviewed the manuscript. YT conceived the study and reviewed the manuscript. All authors contributed to the article and approved it for publication.

## Conflict of Interest

The authors declare that the research was conducted in the absence of any commercial or financial relationships that could be construed as a potential conflict of interest.

## Publisher's Note

All claims expressed in this article are solely those of the authors and do not necessarily represent those of their affiliated organizations, or those of the publisher, the editors and the reviewers. Any product that may be evaluated in this article, or claim that may be made by its manufacturer, is not guaranteed or endorsed by the publisher.
